# How Kentucky bluegrass tolerate stress caused by sodium chloride used for road de-icing?

**DOI:** 10.1007/s11356-018-3640-4

**Published:** 2018-11-12

**Authors:** Grażyna Mastalerczuk, Barbara Borawska-Jarmułowicz, Hazem Mohamed Kalaji

**Affiliations:** 1 Department of Agronomy, Faculty of Agriculture and Biology, Warsaw University of Life Sciences-SGGW, 159 Nowoursynowska St., 02-776 Warsaw, Poland; 2 Institute of Technology and Life Sciences (ITP), Falenty, Al. Hrabska 3, 05-090 Raszyn, Poland; 3 Department of Plant Physiology, Faculty of Agriculture and Biology, Warsaw University of Life Sciences-SGGW, 159 Nowoursynowska St., 02-776 Warsaw, Poland

**Keywords:** Chlorophyll *a* fluorescence, Germination capacity, NaCl, *Poa pratensis*, Tillering

## Abstract

Salts used in road de-icing during winter season inhibit the growth and development of lawn grass species. The mechanism of plant tolerance/sensitivity to such treatments is still not clear. Moreover, there is a lack of fast and non-invasive tool to detect the effect of these salts on plants growth. This study was designed to understand the tolerance mechanism of Kentucky bluegrass plants on salinity, based on some biometric and physiological parameters. In this experiment, we simulated the urban conditions where salts are used intensively for roads de-icing. Germination capacity was evaluated at five salt solutions of NaCl (0, 50, 100, 150 and 200 mM), and physiological parameters were measured during the tillering phase at salinity levels of 0, 150 and 300 mM of NaCl. Seeds of Kentucky bluegrass did not germinate under salinity. During tillering phase, salinity affected length, area and dry mass of roots as well as the relative water content of plants, negatively. Moreover, it influenced the maximum chlorophyll fluorescence yield, quantum yield of photosystem II and electron transport rate at early period of stress. This allows us to recommend these parameters for early detection of soil salinity effects on Kentucky bluegrass plants. It seems to be that the tolerance of this plant towards salinity is based on retaining water content in leaves that allow more efficient functioning of photosynthetic apparatus.

## Introduction

Road de-icing salts are widely used in the northern hemisphere to maintain clear roads in the winter months. The most commonly used substances are sodium chloride (NaCl), calcium chloride (CaCl_2_), and magnesium chloride (MgCl_2_), where in NaCl accounts for 98% of all usage. The application of other methods is not significant, due to the comparatively low cost and high availability of sodium chloride (Czerniawska-Kusza et al. 2004; Mazur 2015). The amount of sodium chloride to de-icing of roads in Poland vary from 15 to 30 g m^−2^ as a single dose (Czarna 2013). For this reason, use of NaCl in the winter time causes soil salinity, which negatively affects the growth and development of plants and soil environment (Wrochna et al. 2010). Plant response to the presence of NaCl in the soil can be varied during germination and subsequent growth stages. Excessive soil salinity limits the development of lawn grasses, which adversely affects their appearance and recreational properties of the turf, especially in poor habitats. Salinity has the greatest impact on the germination of seeds and the growth of grass seedlings due to the accumulation of salt compounds in the surface of the soil. Simultaneously studies concerning the seed germination and the initial period of growth of lawn grasses under salinity are conducted on the basis of different methods as well as light and temperature conditions (Zhang et al. 2011; Zhang et al. 2012; Borawska-Jarmułowicz et al. 2017). According to literature (Alshammary et al. 2004), excessive salinity affects the structure of the root tissue and induced disorders in normal downloading and conduction of the water to the aboveground parts of the plants.

Salt stress causes many disturbances of life processes, including photosynthesis (Acosta-Motos et al. 2017). Matters of the influence of salinity on the functioning of photosystem PSII of plants have often been taken in studies, but the results are not always conclusive. Many of them indicate that salt stress can disrupt the operation of photosystem PSII of various plant species (Jimenez et al. 1997; Stępień and Kłobus 2006; Kalaji and Guo 2008). Reducing the efficiency of PSII system on the light and salinity conditions was observed in tomato plants (He et al. 2009; Zribi et al. 2009) and cucumber seedlings (Zhang et al. 2009). There are also data which indicate a high photochemical activity of PSII system under salinity (Lu et al. 2002). Trait essential to plant function is relative water content (RWC), which determine water status of a shoot relative to its fully hydrated state. Under saline condition plants usually adjust their osmotic potential to maintain turgor pressure. Quantifying the effect of salinity on RWC is important and physiologically relevant (Negrão et al. 2017).

Lawn grasses demonstrate varied tolerance to salinity (Borowski 2008; Borawska-Jarmułowicz et al. 2017). Kentucky bluegrass belongs to the most valuable grasses both fodder and lawn. This species characterised by excellent permanence, slow rate of regrowth, resistance to trampling and tolerates low mowing. Because of the production of many vegetative shoots, this species forms a dense and strong sward (Mastalerczuk et al. 2012; De 2017). Despite its many advantages, Kentucky bluegrass is sensitive to salt stress, and its lawn cultivars can be recommended only as a complementary component in mixtures on soil with a slight salinity (Borowski 2008).

The aim of this study was to evaluate the effect of different levels of NaCl conditions on seed germination and some biometric indicators of lawn cultivar of Kentucky bluegrass. It was also to check if some measured and calculated parameters of photosynthetic performance can be used as bioindicators for early detection of salinity stress effects on Kentucky bluegrass plants. Moreover, we intended to find some tolerance mechanisms based on the changes of relation between various studied parameters.

## Materials and methods

### Plant cultivation and treatment

Studies on the influence of sodium chloride (NaCl) salinity on germination capacity, morphological, biometric parameters and physiological characteristics of Kentucky bluegrass (*Poa pratensis* L.) lawn cv. Sójka were carried out in two separated experiments under controlled conditions: in laboratory (Pol EkoST 600 thermostat) and growth chamber (Phytotron—BLOCK a.s., Valašské Meziříčí, Czech Republic).

Laboratory experiment was established to evaluate germination capacity (%) of tested seeds according to ISTA Rules (2016) and in salt stress conditions. To sterilise the surface of the seeds, they were treated with a 70% solution of ethanol for 2 min and then quickly dried with a tissue paper. Four replicates of 50 seeds of Kentucky bluegrass were placed on 10-diameter Petri dishes laid with two layers of filter paper (grammage 65 g m^−2^). Germination test, according to the ISTA Rules (2016), was carried out in accordance with different temperature and light conditions: 30/20 °C (day/night) temperature regime and 8/16 h (day/night) photoperiod under cool, white light 20 W m^−2^. Seeds were germinated at 60% moisture with distilled water (compared to control volume of the water). Germination capacity in salt stress condition was estimated in constant temperature 20 °C (day/night) and variable photoperiod 12/12 h (day/night) under the same light (20 W m^−2^). Filter paper was moistened in salt solutions (mM NaCl): 0, 50, 100, 150 and 200. The electrical conductivity (EC) measured of each salt solution was 0.0, 5.5, 9.2, 12.8 and 18.0 dSm^−1^, respectively. Data were collected based on the final counts of all normal seedlings after 21 days. The length of shoots and roots of seedlings and their dry mass were determined. The ratio of root to shoot dry mass (R:S) was also estimated. The obtained results were expressed per seedling.

In the second experiment (growth chamber), the study was conducted in plastic pots of 150 mm in diameter and 180 mm in height. The pots were filled with podzolic soil formed from loamy sand, which was collected from the upper 20 cm of sand at the Experimental Station farm field of the Warsaw University of Life Sciences-SGGW. Concentrations of available soil phosphorus were 94.5 mg kg^−1^, potassium 99.6 mg kg^−1^ and magnesium 40.0 mg kg^−1^. Soil pH_KCl_ was 5.2. The soil in each pot was mixed with 0.13 g N, 0.15 g P and 0.15 g K. During study period, the soil moisture was maintained at 70% of capillary water capacity by watering with distilled water every 2 days to a given weight of the soil and pot.

Growth conditions of plants in a growth chamber were set and automatically controlled during the study period. A relative humidity of air was 65%, the photoperiod for the day/night cycle was 16/8 h and radiation during the day was 95 (W m^−2^) (350 PAR). The temperature was gradually increased (2 °C per hour) from 12 °C at night to 26 °C during the day. The maximum temperature (26 °C) was maintained for 4 h and then gradually reduced to 12 °C.

Three seeds of Kentucky bluegrass were sown in four locations per pot. Directly after the germination phase, redundant seedlings were removed and the research was continued on four plants in a pot. The plants were grown under stress-free conditions until the tillering phase (40 days after sowing), and then three levels of solution of salt concentrations (mM NaCl) were applied: 0, 150 and 300 (EC: 0.0, 12.8 and 18.0 dS m^−1^, respectively). Treatment applications were used in an amount of 150 ml per pot every 4 days. Plants were exposed to different salinity treatments for 21 days.

Biometric features as well as physiological parameters were evaluated in four terms: after 7, 14, and 21 days of NaCl application and after 35 days (2 weeks after withholding salt stress application). The experiment was designed as a completely randomised plot with three replicates for each treatment and term of measurement.

### Biometric parameters of plants

The shoots number per plant were evaluated and then the plants were cut at tillering nodes. Soil with roots from the pots (in three replications for treatment) in each term was sieved with a sieve having a mesh size of 3.0–0.3 mm. The roots of plant were washed in a gentle stream of water to separate them from the soil. The collected root material was scanned by an optical scanner Epson Perfection V700 Photo (resolution 400 pdi) and converted to morphometric analysis using software WinRhizo 2012 (Regent Instruments Inc., Canada). The total root length (cm per plant), average diameter (mm) of roots and roots area per plant (cm^2^) were evaluated. Plant samples were dried at 105 °C for 24 h (consistent with weight) and the shoot and root dry mass (g per plant) were determined. These data were used to calculate the root-to-shoot ratio (R:S) according to the formula (Monk 1966):

$$ \mathrm{R}:\mathrm{S}=\frac{\mathrm{dry}\ \mathrm{mass}\ \mathrm{of}\ \mathrm{roots}}{\mathrm{dry}\ \mathrm{mass}\ \mathrm{of}\ \mathrm{shoots}} $$

Based on the total root length and their dry mass data, the specific root length (SRL; m g^−1^) was calculated (Ostonen et al. 2007):

$$ \mathrm{SRL}=\frac{\mathrm{total}\ \mathrm{root}\ \mathrm{length}}{\mathrm{dry}\ \mathrm{mass}\ \mathrm{of}\ \mathrm{root}\mathrm{s}} $$

### Physiological parameters of plants

Measurements of selected chlorophyll *a* fluorescence parameters using fluorometer FMS-2 (Modulated Chlorophyll Fluorescence System - Hansatech Instruments Ltd., UK) were done. The quantum efficiency of photosystem II (Φ_PSII_), the maximal fluorescence signal (F_*m*_′) and steady-state (F_*s*_) chlorophyll fluorescence yields of light-adapted samples were measured. Measurements were performed on each plant in the middle of the fully developed leaf blades. Chlorophyll fluorescence parameters are shown as relative units. The photosynthetic electron transport rate (ETR) was calculated as (Ralph et al. 2005):

$$ ETR=Y\times PAR\times 0.5\times 0.84 $$

where Y represents the quantum yield of photosystem II (PSII) electron transport (Φ_PSII_); (PAR) photosynthetically active radiation - measured automatically by a fluorometer; 0.5 represents the fact that an electronic transmission needs to absorb two photons, assuming that the light energy absorbed by a photosynthetic system can be distributed to the photosynthetic system PSI and PSII by the same proportion, i.e. 50% each; 0.84 is the absorption coefficient indicating that a light energy incidence of only 84% can be absorbed by the leaves.

To estimate relative water content (RWC; %), the leaves of Kentucky bluegrass plants were cut and their fresh mass was determined. Then, they were submerged for 12 h in distilled water at room temperature (20 °C) to determine their turgid mass. The RWC was calculated from the equation (Turner 1981):

$$ \mathrm{RWC}=\frac{\mathrm{leaf}\ \mathrm{fresh}\ \mathrm{mass}\hbox{--} \mathrm{leaf}\ \mathrm{dry}\ \mathrm{mass}}{\left(\mathrm{turgid}\ \mathrm{mass}\hbox{--} \mathrm{leaf}\ \mathrm{dry}\ \mathrm{mass}\right)\times 100\%} $$

### Statistical analysis

Results of the influence of salinity levels on biometric-morphological features and physiological parameters were compiled by statistically two-way analysis of variance using software Statistica 12.5 (Statsoft, Inc. Tulsa, USA). Tukey’s test was used as post hoc test with *a* = 0.05 to determine the significance of differences between the means. Relations between the studied traits and their variation in salinity conditions were determined on the basis of the principal components analysis (PCA) and by correlation analysis.

## Results

The germination capacity of the Kentucky bluegrass cv. Sójka seeds significantly depended on the applied NaCl levels. It was found that in salinity conditions, the seeds did not germinate (even at 50 mM NaCl) (Table 1). The obtained results also showed that the seeds germinated better on medium saturated with distilled water under variable temperature and light conditions (91.8%) than at constant temperature and 12 h photoperiod (25.2%). In spite of this morphological parameters of seedlings were similar in both conditions.

**Table 1:** Influence of NaCl solution on selected germination parameters of Kentucky bluegrass cv. Sójka

Germination conditions			Germination capacity	Lenght		Dry mass		
Temperature	Day/night	NaCl		Leaves	Roots	Leaves	Roots	R/S ratio
(°C)	(h)	(mM)	(%)	(mm)		(mg)		
30/20	8/16	0	91.8^c*^	36.80^b^	45.18^b^	0.67^b^	0.05^b^	0.07^b^
20/20	12/12	0	25.2^b^	30.50^b^	35.25^b^	0.58^b^	0.04^b^	0.07^b^
20/20	12/12	50	0.0^a^	0.00^a^	0.00^a^	0.00^a^	0.00^a^	0.00^a^
20/20	12/12	100	0.0^a^	0.00^a^	0.00^a^	0.00^a^	0.00^a^	0.00^a^
20/20	12/12	150	0.0^a^	0.00^a^	0.00^a^	0.00^a^	0.00^a^	0.00^a^
20/20	12/12	200	0.0^a^	0.00^a^	0.00^a^	0.00^a^	0.00^a^	0.00^a^

*Mean values in the columns marked with the same letters do not differ significantly according to Tukey’s HSD at *p* ≤ 0.05

There was no impact of salinity on the number of shoots of Kentucky bluegrass in the tillering phase (Table 2). The effect of NaCl on the aboveground part of plant was visible only after 21 days of salinity application. Both of NaCl solutions (150 and 300 mM) resulted in decrease of fresh and dry mass of plants. In the later period (35th day), the aboveground mass of plants reached similar values at all levels of salinity, while there was a reduction in the number of shoots on plant under NaCl conditions.

**Table 2:** Effect of NaCl concentration (0, 150, 300 mM) on the number, fresh and dry mass of shoots, dry mass, length, average diameter and area of roots, specific root length and the root to shoot ratio of the Kentucky bluegrass plants cv. Sójka in terms of measurements (7, 14, 21 and 35 days from NaCl application) in the tillering phase

Features	NaCl (mM)	Days				
		7	14	21	Mean 7–21	35
Shoots No. (No. plant^−1^)	0	7.3^a*^	16.2^a^	20.7^a^	14.7^a^	28.3^b^
	150	7.5^a^	12.7^a^	19.0^a^	13.0^a^	16.8^a^
	300	8.7^a^	14.0^a^	17.3^a^	13.3^a^	16.8^a^
Shoots fresh mass (g plant^−1^)	0	2.06^a^	3.92^a^	5.51^b^	3.83^b^	6.30^a^
	150	1.83^a^	2.99^a^	3.56^a^	2.79^a^	5.90^a^
	300	1.68^a^	2.79^a^	3.44^a^	2.64^a^	5.98^a^
Shoots dry mass (g plant^−1^)	0	0.42^a^	0.87^a^	1.51^b^	0.94^b^	2.22^a^
	150	0.38^a^	0.70^a^	0.98^a^	0.69^a^	2.08^a^
	300	0.36^a^	0.74^a^	1.03^a^	0.71^a^	2.17^a^
Roots dry mass (g plant^−1^)	0	0.11^b^	0.25^a^	0.42^b^	0.26^b^	1.22^b^
	150	0.08^ab^	0.17^a^	0.34^b^	0.20^b^	0.99^b^
	300	0.07^a^	0.22^a^	0.25^a^	0.18^a^	0.55^a^
Roots length (cm plant^−1^)	0	2237^b^	5014^ab^	11136^b^	6129^b^	23526^b^
	150	1814^a^	4061^a^	7058^a^	4311^a^	21845^b^
	300	1883^a^	5603^b^	7206^a^	4897^a^	13541^a^
Average roots diameter (mm)	0	0.77^c^	0.73^b^	0.61^a^	0.70^b^	0.61^a^
	150	0.67^b^	0.69^b^	0.74^b^	0.70^b^	0.59^a^
	300	0.60^a^	0.58^a^	0.71^b^	0.63^a^	0.66^b^
Roots area (cm^2^ plant^−1^)	0	483^b^	1246^c^	1867^b^	1199^b^	4222^b^
	150	380^a^	876^a^	1659^a^	972^a^	4007^b^
	300	353^a^	1029^b^	1581^a^	988^a^	2590^a^
Specific root length (m g^−1^)	0	197^a^	203^a^	264^ab^	221^a^	192^a^
	150	224^ab^	236^a^	207^a^	222^a^	224^a^
	300	256^b^	260^a^	299^b^	272^b^	254^b^
Root to Shoot ratio	0	0.26^b^	0.29^a^	0.29^b^	0.28^b^	0.56^b^
	150	0.21^a^	0.25^a^	0.35^b^	0.27^b^	0.49^b^
	300	0.21^a^	0.30^a^	0.25^a^	0.25^a^	0.26^a^

*Mean values in the columns marked with the same letters do not differ significantly according to Tukey’s HSD at *p* ≤ 0.05

The analysis of ANOVA showed that some of roots parameters were significantly affected by the levels of salinity (Table 2). The application of NaCl, especially 300 mM, caused a decrease in the dry mass of the plant roots. A lower root proportion in root to shoot ratio under salt stress was also stated. This reaction was visible not only during the application of NaCl, but also after its withholding (35th day). The length and area of roots were the highest under controlled conditions during the stress, while after 2 weeks (35th day), these parameters were similar both 0 and 150 mM NaCl. The average diameter of plants roots was reduced by the applying of NaCl in the first 14 days. In the next period, this parameter increased and was higher compared to the values obtained under control conditions. The absence of salt to plants that have been previously treated with 300 mM NaCl solution has promoted the development of thicker roots. The results also showed that plants under salinity condition characterised by higher SRL than under controlled. This dependence persisted also after withholding NaCl application.

The response of Kentucky bluegrass physiological parameters to salt conditions was differentiated during the study period (Table 3). The values of F_*m*_′ were significantly lower in salinity conditions at each term during NaCl application, while the reduction of F_*S*_ was visible also in the subsequent measurement term (35th day). It was found that applied salt solutions had significant effect on Φ_PSII_ and ETR. Higher Φ_PSII_ and ETR were observed in plants growing under control conditions (0 mM NaCl). The results also showed a reduction in RWC under NaCl application. Lower RWC of plant leaves under the influence of increasing salt solutions was observed in all terms of measurements.

**Table 3:** The effect of NaCl concentration (0, 150, 300 mM) on steady-state (F_*S*_) and maximal (F_*m*_′) chlorophyll *a* fluorescence yields of light-adapted samples, quantum yield of PSII electron transport (Φ_PSII_), photosynthetic electron transport rate (ETR) and relative water content (RWC) of Kentucky bluegrass plants in terms of measurements (7, 14, 21 and 35 days from NaCl application) in the tillering phase; chlorophyll fluorescence parameters are shown as relative units

Features	NaCl (mM)	Days				
		7	14	21	Mean 7–21	35
F_S_	0	770^b*^	815^a^	895^b^	827^b^	770^b^
	150	717^ab^	859^a^	707^a^	761^a^	708^ab^
	300	683^a^	790^a^	728^a^	734^a^	648^a^
F_m_′	0	2797^c^	3005^b^	2781^b^	2861^b^	1861^a^
	150	2555^b^	2684^a^	2309^a^	2516^a^	1848^a^
	300	2204^a^	2524^a^	2420^ab^	2383^a^	1750^a^
Φ_PSII_	0	0.722^b^	0.728^b^	0.693^a^	0.714^b^	0.684^b^
	150	0.716^b^	0.676^a^	0.695^a^	0.697^ab^	0.642^ab^
	300	0.691^a^	0.685^a^	0.699^a^	0.691^a^	0.629^a^
ETR	0	104.1^b^	104.0^b^	98.9^b^	102.6^b^	101.9^b^
	150	103.3^ab^	95.7^a^	99.0^b^	99.3^b^	85.3^a^
	300	99.6^a^	96.9^a^	92.3^a^	96.3^a^	83.5^a^
RWC (%)	0	91.5^b^	92.3^b^	87.8^b^	90.5^b^	86.6^c^
	150	88.9^ab^	85.8^a^	80.6^a^	85.1^a^	77.2^b^
	300	86.0^a^	84.0^a^	80.4^a^	83.5^a^	71.7^a^

*Mean values in the columns marked with the same letters do not differ significantly according to Tukey’s HSD at *p* ≤ 0.05

## Discussion

The road de-icing NaCl which was applied in our study was found to be influencing adversely on the treated plants but this effect varied depending on growth stage (germination and tillering), salt concentration and duration of stress.

Salinity influences seed germination primarily by lowering the osmotic potential of the soil solution to delay water absorption by seeds, causing sodium and/or chloride toxicity to the embryo or by altering protein synthesis (Kalaji and Pietkiewicz 1993). The obtained results showed that seed germination of Kentucky bluegrass was highly affected by applied levels of salinity (Table 1). Under salt stress, seeds did not germinate even at a lower concentration of NaCl (50 mM NaCl). Based on literature (Guesdon et al. 2016), this concentration is considered as mild saline solution. The sensitivity of this species during the germination on the presence of NaCl in the medium, previous studies have also shown (Friell et al. 2013; Zhang et al. 2013; Borawska-Jarmułowicz et al. 2017). Results of Zhang et al. (2013) and Nizam (2011) both point to the negative effect of increasing concentrations of NaCl in subsoil on dry mass, morphological characteristics as well as the number of roots of turfgrass seedlings. In the study of Borawska-Jarmułowicz et al. (2017), salinity increased also mean germination time of the tested grass species. Poor seed germination of Kentucky bluegrass under conditions 0.0 Mm NaCl could be result of lower and constant temperature (20 °C) used at laboratory test compare to varied temperature and light conditions applied at germination according to ISTA Rules (2016).

We found that salinity had small effect in the initial period of NaCl application on number of shoots, as well as fresh and dry mass of plants during tillering phase (Table 2). The reduction of aboveground mass of plants was found only after 21 days of salinity, although according to literature (Czarna 2013; Guesdon et al. 2016) concentrations of NaCl used in the study are considered as hypersaline solutions. The winter season in Poland begins on October 15 of a given year and continues until April 14 of the following year. The actual duration and the number of announced actions to keep the streets clear of ice and snow depend on the weather conditions and the state of the street surfaces. In urban conditions of central Poland public demand for proper winter maintenance of streets, which includes salting truck actions coincides with snowy winters, such as those in the year 2004/2005, there were 50 events of salt application, in 2009/2010–53 salt applications, or 45 during last winter—2017/2018 (data from Zarząd Oczyszczania Miasta—ZOM, Department of Mechanical Cleaning—Streets 2018). According to Czarna (2013), a single application of NaCl should not exceed 30 g m^–2^, so for the above data the total seasonal dose of salt range from 1350 to 1590 g m^–2^. In our studies, applied single dose was higher and with solution of 150 mM NaCl was 74.6 g m^–2^, and with 300 mM NaCl—148.7 g m^–2^. However, the total amount of salt was less and reached 447.6 and 892.4 g m^–2^, respectively. The obtained results indicate that the applied high NaCl doses negatively affected the aboveground mass of Kentucky bluegrass plants only after 21 days, while root mass was limited only with higher salinity (about 149 g m^–2^).

Accumulation of Na^+^ ions turns out to be toxic especially in older leaves, which are no longer expanding and so no longer diluting the salt arriving in them as young growing leaves do (Parihar et al. 2015). If the rate at which they die is greater than the rate at which new leaves are produced, the photosynthetic capacity of the plant will no longer be able to supply the carbohydrate requirement of the young leaves, which further reduces their growth rate (Munns and Tester 2008). In our study delay in premature senescence of old leaves (carbon source) of Kentucky bluegrass could be a symptom of tissue adaptations to salinity. Cushman et al. (1990) stated that the most sensitive to salinity are the youngest cells of shoots and roots in the intensive growth phase. According to many authors (Wilson et al. 1992; Kalaji and Pietkiewicz 1993) root growth under salinity conditions is generally less inhibited than growth of the aboveground part of plant. The root system is responsible for water uptake, accompanied by dissolved ions (including Na^+^), and thus plays an essential role in preventing Na^+^ from entering the vascular system and reaching the shoot. Salinity stress is first perceived by the roots and impairs plant growth both in the short term, by inducing osmotic stress caused by reduced water availability, and in the long term, by salt-induced ion toxicity due to nutrient imbalance in the cytosol (Acosta-Motos et al. 2017). The roots are the organs which, as a defence mechanism, can limit the negative effects of excess ions in the medium by their secretion or accumulation. There are also studies showing differentiated root responses to salinity in species sensitive and tolerant. In the study of Nieman et al. (1988) there were found that the root growth in pepper plants (sensitive plant) was inhibited while the plants of sunflower (resistant plant) did not respond to salinity. Simultaneously, in both species, limited growth of the aboveground part was observed. The increase in root mass due to the salinity of resistant species of grasses also stated Gulzar et al. (2003) and Hameed and Ashraf (2008). While, Małuszyńska and Małuszyński (2009) in their studies of Cocksfoot and Italian ryegrass were found inhibitory impact of increasing soil salinity on both of plant parts: above- and belowground. Our study showed significant differences in biometric characteristics of the roots of plants under the conditions of salinity levels (Table 2). Roots of Kentucky bluegrass accumulated less dry mass under the influence of salt stress. Similarly, the root to shoot ratio (R:S) under NaCl was lower than that of control (0.0 mM NaCl) and this relationship persisted also after withholding salt application. According to Cassaniti et al. (2009), a greater root proportion in root to shoot ratio under salt stress can favour the retention of toxic ions in this organ, controlling their translocation to the shoots. This response can constitute a typical mechanism of plant resistance/survival under saline conditions.

In our study, negative influence of NaCl on the growth of Kentucky bluegrass plants was also found in relation to the length and surface area of roots. After 3 weeks of salt stress (21st day) values of these parameters in the control plants were significantly higher than those obtained under NaCl salinity conditions (150 and 300 mM). Simultaneously, the withholding of NaCl stress application, within 14 days (35th day), reduced the length and surface area of roots of plants previously grown under higher salinity conditions (300 mM NaCl). The reduction of the dry mass of shoots and roots, and the length of roots of lawn grass cultivars, including Kentucky bluegrass, under the influence of increasing concentrations of NaCl was also recorded by Zhang et al. (2013). The authors found a stronger negative reaction of the belowground part of the plant (mass and length of roots) compared to aboveground (shoots mass).

The highest concentration of NaCl (300 mM) also influenced the SRL of Kentucky bluegrass plants (Table 2). High SRL demonstrate a greater share of fine roots of smaller diameter in the conditions of salinity. According to Ostonen et al. (2007) SRL is an indicator that describes well the reactions of plants to changes in the soil environment. The higher value of SRL, the better the branching of the roots, allowing the plants to survive periods of water or nutrient deficiencies (Hill et al. 2006). In the study of Rubinigg et al. (2003) SRL of *Plantago maritima* plants also increased upon exposure to elevated sodium chloride concentrations due to variations in biomass allocation and length growth of the tap root. In the Carillo et al. (2011) study apical region of roots grown under salinity showed extensive vacuolization and lack of typical organisation of apical tissue. Authors stated a slight plasmolysis due to a lack of continuity and adherence between cells with a tendency to the arrest of growth and differentiation in salinity conditions, while control plants root tips are characterised by densely packed tissues with only small intercellular spaces.

Chlorophyll fluorescence quenching analysis has proven to be a reliable method to estimate the changes in function of photosystem II (PSII) under varied stress conditions (Kalaji et al. 2016). In this experiment, the influence of salt stress on the functioning of Kentucky bluegrass plant photosynthetic apparatus also was found in relation to all measured chlorophyll *a* fluorescence parameters (Table 3). The influence of applied high salinity on the evaluated physiological parameters was visible clearly earlier than on the morphological traits of plants. Already after 7 days of using both NaCl solutions, smaller values of all tested parameters were found. This was also evident after 3 weeks of applying the salinity conditions. According to Sudhir and Murthy (2004), salt stress increases the accumulation of NaCl in chloroplasts of higher plants, affects growth rate, and is often associated with decrease in photosynthetic electron transport activities in photosynthesis. The accumulation of intracellular sodium ions at salinity conditions changes the ratio of K:Na, which affect the bioenergetic processes of photosynthesis. In our study salinity also decreased Φ_PSII_ and ETR. Acosta-Motos et al. (2017) reported that under salinity decreases in PSII efficiency and increases in non-photochemical quenching parameters as a mechanism to safely dissipate excess energy. In the study of Moradi and Ismail (2007) salt stress was found to reduce ETR in a salt-sensitive rice cultivar, whereas only a slight reduction in ETR occurred in a salt-tolerant cultivar. The study conducted by Kalaji et al. (2011) on barley also showed the influence of salinity stress on chlorophyll fluorescence parameters. Application of NaCl (120 mM) resulted in significant reduction in values of F_*m*_′, Φ_PSII_ and ETR. In our study, reduction in values of Φ_PSII_ and ETR were recorded not only at NaCl application but also after its withholding. This indicates the possibility of early and non-invasive detection of changes in the functioning of the photosynthetic apparatus of plants using the parameters evaluated under salt stress conditions. Bai et al. (2017) suggests that long-term salt treatments limit photochemical conversion efficiency of PSII, destroy the PSII reaction center, and next inhibit leaf growth of plant.

Kentucky bluegrass plants are also characterised by significantly lower RWC in leaves under salinity. Decrease RWC, for both NaCl concentration levels, was observed at each term, and even at 35th day, i.e. 2 weeks after the salt application ceased. Lower RWC of leaves under the influence of increasing concentrations of NaCl was also recorded by Uddin et al. (2012) in the study on lawn grasses. Whereas Ziaf et al. (2009) proposed the RWC parameter as a reliable indicator of salinity tolerance in plants of pepper.

**Fig. 1:**
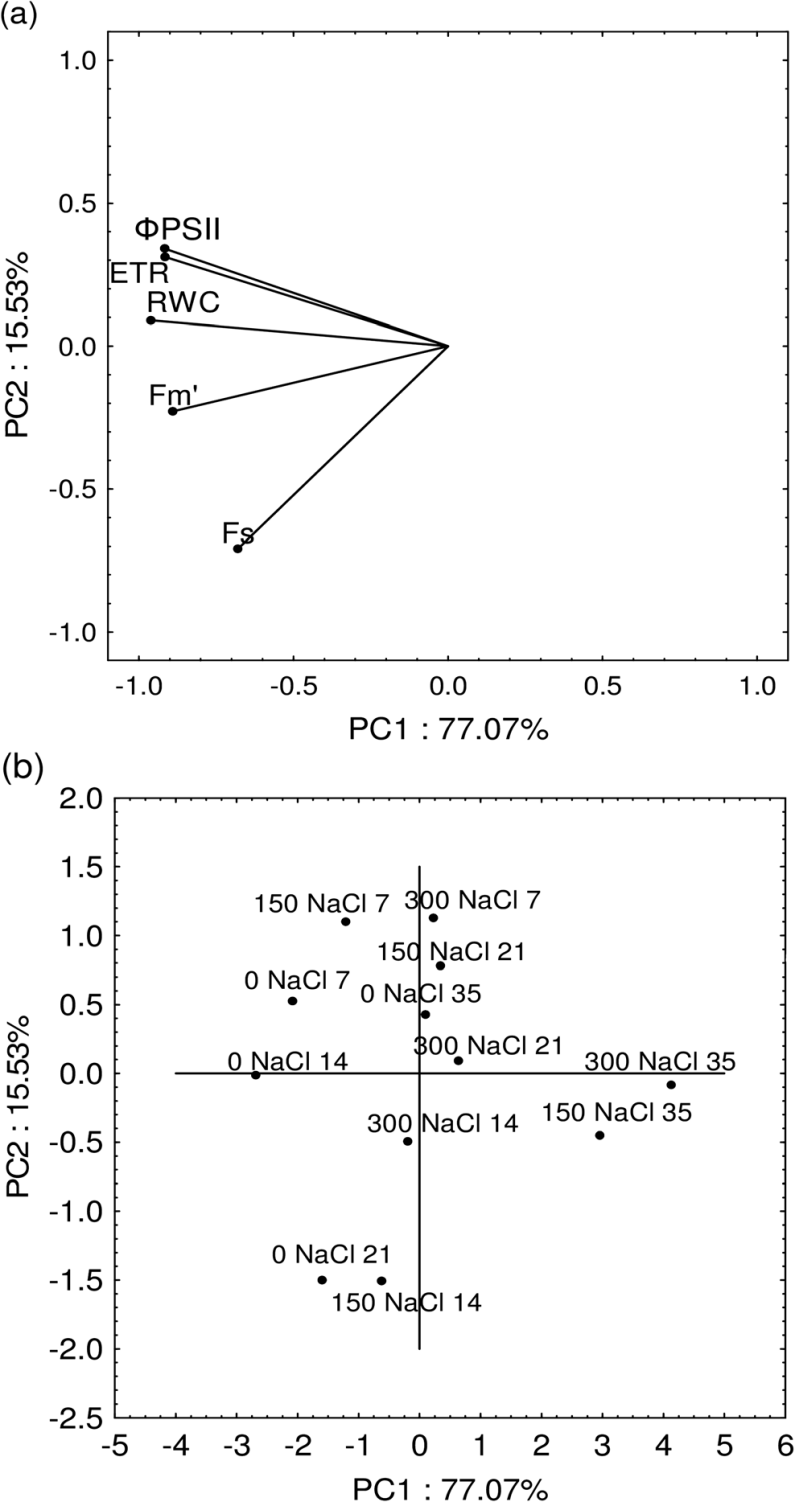
The values of principal components PC1 and PC2 (**a**) for the chlorophyll fluorescence parameters studied: F_*S*_, F_*m*_′, Φ_PSII_, ETR and RWC; (**b**) for the combinations tested (levels of salinity: - 0, 150 and 300 mM NaCl and terms of measurements - 7, 14, 21 and 35 days from NaCl application)

PCA analysis showed that the first component accounted for 77.01% and the second 15.53% of the analysed variability (Fig. 1). The salinity of the Kentucky bluegrass plants most affected RWC, Φ_PSII_, ETR and to a less extent F_*m*_′. These parameters were significantly, positively correlated with each other, while F_*S*_ was independent variable (Fig. 1a). The lowest values of these characteristics at both salinity levels (150 and 300 mM NaCl) were recorded at day 35, that is until 2 weeks after withholding the application of NaCl (Fig. 1b).

**Fig. 2:**
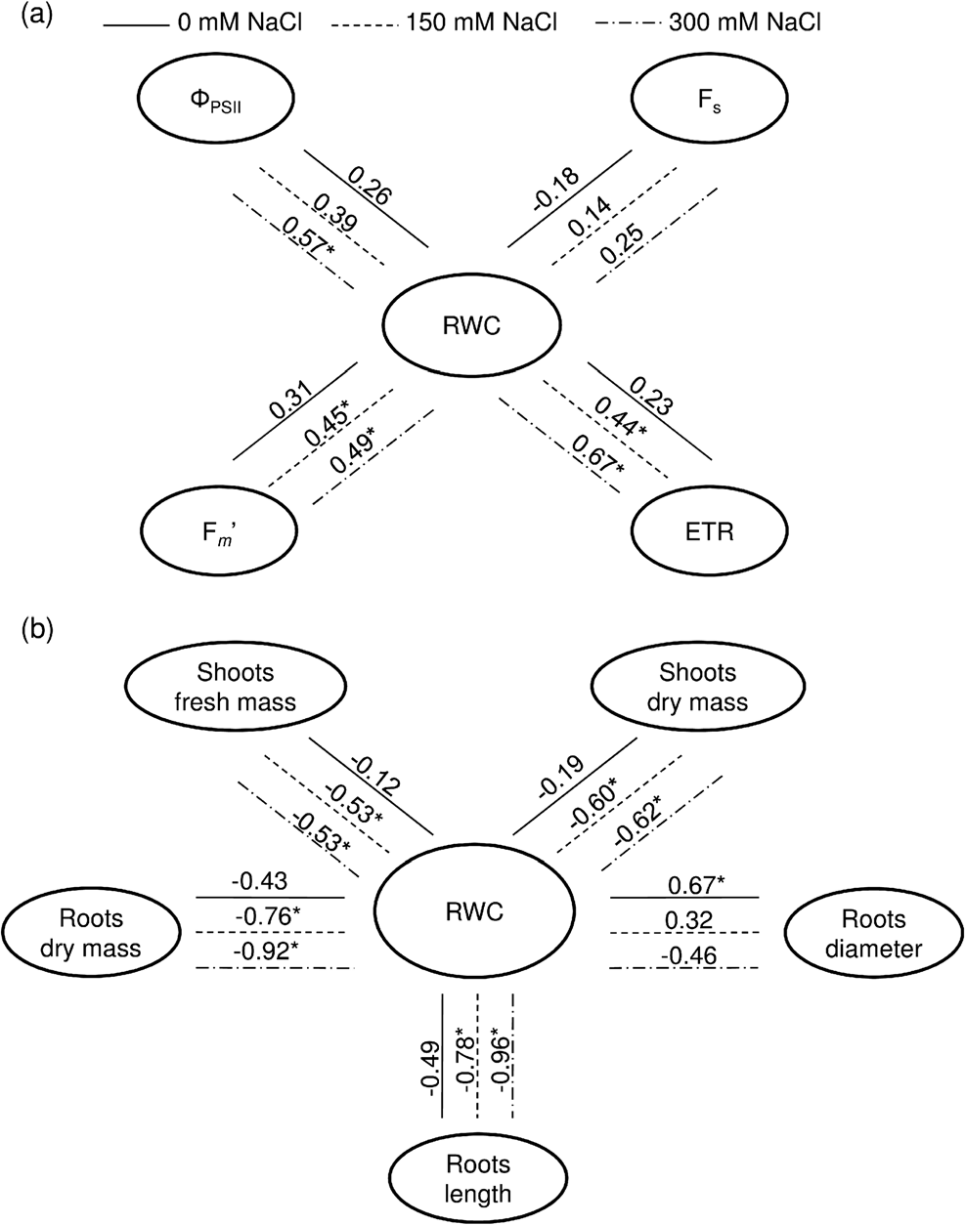
Correlation coefficients (*r*) between relative water content (RWC) and (**a**) selected physiological parameters: quantum yield of PSII electron transport (Φ_PSII_), steady-state (F_*S*_) and maximal (F_*m*_′) chlorophyll *a* fluorescence yields of light-adapted samples, photosynthetic electron transport rate (ETR) and (**b**) biometric traits; *correlations significant at *p* ≤ 0.05

Comparison of correlation coefficients between RWC and selected physiological and biometric characteristics allowed to analyse different adaptation strategies of Kentucky bluegrass plants under control conditions and under salinity. In the absence of salinity (0 mM NaCl) plants did not show significant correlations between these groups of characteristics, while there were significant relations between them under both levels of salt solution (except Φ_PSII_, which was significant at 300 mM NaCl) (Fig. 2). Based on comparison of the obtained in this work results with our previous published work (Mastalerczuk et al. 2017), we assume that the effect of salinity on the photosynthetic efficiency of this plant is rather due to water stress than toxic effects. That was confirmed by the strong correlations between relative water content and the measured photosynthetic efficiency parameters.

Our results revealed that, plant water content was the decisive factor behind keeping the photosynthetic functioning of the plants. That was underlined by the strong correlations between RWC and F_*m*_′, Φ_PSII_, ETR (Fig. 2a). Kalaji and Pietkiewicz (1993) suggested that under conditions of salt stress plants regulate photosynthetic processes to survive adverse conditions. In our study, mechanism was verified by the strengthened relationship between the majority of the surveyed parameters (significant correlation coefficients). Similar dependence was observed in the case of biometric parameters. RWC of Kentucky bluegrass was significantly linked with biometric traits (except roots diameter) under both levels of NaCl salinity. This suggests that plants with lower RWC have more biomass under saline and therefore more efficiently use water under stress conditions (Fig. 2b).

## Conclusions

Our results revealed that the studied cultivar Sójka of Kentucky bluegrass is very sensitive to salinity due that its seeds did not germinate under solution of any applied NaCl level. During the tillering phase, the application of salinity stress (150 and 300 mM NaCl) did not affect the number of shoots, while fresh and dry mass of aboveground parts of plants changed only at the end of the stress application. This can indicate to a good adaptation of plants to NaCl within the range of the used concentrations during this growth phase. Root mass as well as their biometric characteristics (length and surface) and also root to shoot ratio (R:S) were lower in plants grown under conditions of applied NaCl concentrations, especially under 300 mM as compared to control ones. The specific root length (SRL) was higher under application of 300 mM NaCl. That points to a greater proportion of smaller roots diameter at salinity conditions. Applied NaCl concentrations on plants in the tillering phase affected the quantum yield of PSII electron transport (Φ_PSII_), electron transport rate (ETR) and maximal fluorescence signal (F_*m*_′) of light-adapted samples already in the early period of stress, what allow us to recommend these parameters for early detection of salinity conditions effects on Kentucky bluegrass plants. We conclude that Kentucky bluegrass cv. Sójka can tolerate salinity by holding water content in leaves at a level that assure more efficient functioning of photosynthetic apparatus.
